# Alterations in CNS-derived blood biomarkers during 30 days simulated microgravity

**DOI:** 10.3389/fphys.2025.1600708

**Published:** 2025-06-24

**Authors:** Susanne V. Schmidt, Alexandru Odainic, Benjamin Aretz, Petra Frings-Meuthen, Jan-Niklas Hoenemann, Maria Bohmeier, Christian Liemersdorf, Edwin Mulder, Stefan Moestl, Karsten Heusser, Jens Tank, Jens Jordan, Laura de Boni

**Affiliations:** ^1^ Institute for Clinical Chemistry and Clinical Pharmacology, University Hospital Bonn, Bonn, Germany; ^2^ Department of Microbiology and Immunology, Peter Doherty Institute of Infection and Immunology, University of Melbourne, Melbourne, VIC, Australia; ^3^ Institute for Medical Biometry, Informatics and Epidemiology (IMBIE), University of Bonn, Bonn, Germany; ^4^ Institute of Aerospace Medicine, German Aerospace Center (DLR), Cologne, Germany; ^5^ Department of Internal Medicine III, Division of Cardiology, Pneumology, Angiology, and Intensive Care, University of Cologne, Cologne, Germany; ^6^ Chair of Aerospace Medicine, Medical Faculty, University of Cologne, Cologne, Germany

**Keywords:** head down tilt bed rest, CNS, biomarker, neurons, glia

## Abstract

**Introduction:**

Spaceflight induces physiological adaptations, including headward fluid shifts that may impact the central nervous system (CNS). Ground-based analogs such as 6° head-down tilt bed rest (HDTBR) provide a controlled setting to study these neurophysiological effects and assess CNS biomarkers. We therefore analyzed neurological function and CNS-derived blood biomarkers during four NASA-backed 30-day strict HDTBR campaigns to better understand neurophysiological responses to prolonged fluid redistribution.

**Methods:**

Forty participants (18 women, 22 men; mean age ∼36 years) were assigned to different countermeasure groups: lower body negative pressure (LBNP), cycling in HDT followed by wearing thigh cuffs, upright sitting (positive control), and HDTBR without countermeasures. Neurological exams and blood biomarker analyses (SIMOA Quanterix) were performed to assess NfL, GFAP, Aβ_40_, Aβ_42_, and total tau.

**Results:**

No clinical neurological impairments were observed. The LBNP group exhibited robust increases in amyloid-related biomarkers during HDTBR, which persisted into the recovery period. Analysis of within-subject changes over time revealed additional effects. In the cycling and cuffs group, NFL levels increased progressively throughout the study. In the control group, GFAP levels rose gradually, indicating mild glial activation. Tau protein also increased in the LBNP group but returned to baseline levels during the recovery phase.

**Discussion:**

These findings highlight subtle but biologically relevant CNS-related changes in response to different countermeasure strategies during 30-day strict HDTBR. LBNP may enhance metabolite clearance, as reflected in increased Aβ washout. These findings support the use of LBNP as a potential countermeasure to protect brain health during spaceflight and analog missions.

## 1 Introduction

Astronautical spaceflight exposes the nervous system to various stressors, affecting, both, macro- and microstructures of the brain. Magnetic resonance imaging studies showed changes post-flight like altered cerebrospinal fluid dynamics, ventricular volume increases, and gray and white matter alterations, impacting regions related to sensorimotor, visual, and cognitive functions (Barisano et al., 2022; Marshall-Goebel et al., 2021; Roy-O'Reilly et al., 2021; Faerman et al., 2023). Functional changes include decreased connectivity in areas crucial for vestibular processing and cognitive control (Roy-O'Reilly et al., 2021; Faerman et al., 2023).

In this context, analyzing neural and glial biomarkers in serum or plasma in astronauts could add information on the neurological effects of space travel. Studies have revealed alterations in biomarkers related to neurodegeneration, neuroinflammation, oxidative stress, and synaptic plasticity following space missions or in older individuals using space analogs such as 6° head-down tilt bed rest (HDTBR) (Blaber et al., 2023). For example, extended space missions showed notable elevations in serum-derived neurofilament light chains (NfL), suggesting axonal breakdown, and glial fibrillary acidic protein (GFAP), indicating astrocyte activation, while total tau levels decreased after return to Earth (Eulenburg et al., 2021). Interestingly, serum β-amyloid 1-40 (Aβ_40_) and Aβ_42_ increased significantly postflight after long duration space missions (Eulenburg et al., 2021). Previous studies using simulated microgravity have also demonstrated that individual factors such as age, can significantly influence neurobiological responses (Blaber et al., 2023).

To date, investigations of central nervous system (CNS) alterations in spaceflight and analog studies have relied exclusively on blood-based biomarkers (Blaber et al., 2023; Eulenburg et al., 2021). While cerebrospinal fluid (CSF) offers the most direct insight into brain health, no comprehensive studies have been published in astronauts or analog settings, likely due to the invasiveness of lumbar puncture and the need for trained clinical staff. Recent advances in assay technology now allow for the sensitive detection of CNS-related biomarkers in serum or plasma. The choice between these two matrices is a critical aspect of study design, as differences in preparation and protein composition can influence biomarker quantification and impact comparability across studies and between intervention and control groups.

Building on these existing findings, HDTBR serves as a well-established model to simulate key physiological effects of microgravity, including cephalad fluid shifts, cardiovascular deconditioning, and musculoskeletal unloading. By inducing these spaceflight-like adaptations in a controlled environment, HDTBR enables the investigation of neurophysiological changes and the evaluation of potential countermeasures. However, the impact of 30-day HDTBR in individuals up to 55 years old and countermeasure reversing pathological cephalad fluid shifts on neuronal biomarkers remain unclear. We hypothesized that simulated microgravity conditions induce measurable changes in blood concentrations of neural and glial biomarkers (NfL, total tau, GFAP, Aβ_40_, and Aβ_42_), reflecting neurophysiological adaptations to gravitational unloading.

## 2 Methods

### 2.1 Study design

Full methodological details are available in the published methods paper (Moestl et al., 2025). Briefly, this study was part of the Spaceflight-Associated Neuro-Ocular Syndrome Countermeasures (SANS CM) study, a collaboration between NASA and the German Aerospace Center (DLR). Conducted at DLR’s medical research facility in Cologne. The study included four campaigns (September 2021–July 2023) with 47 participants in total, staying for 59 days (15-day baseline, 30-day HDTBR, and a 14-day recovery period). Participants were assigned to one of four groups: (i) lower body negative pressure (LBNP, minus 25 mmHg, 6 h/day), (ii) cycling in a 6° HDT position (60 min) followed by venous constrictive thigh cuffs (50 mmHg, 6 h, 6x/week), (iii)) a positive control group with 6 h/day upright sitting, and (iv) a control group without countermeasures. Strict 6° HDT conditions were enforced, with continuous video monitoring for compliance and physiotherapy on every other day. Participants followed a controlled, precisely measured diet tailored to energy needs. In this study, we focused on secondary endpoints in a subset of participants, including neurological evaluations and blood biomarker analyses related to neurodegeneration.

The study was approved by ethics committees in Germany (Ärztekammer Nordrhein, 2020211) and the United States (IRB at Johnson Space Center, STUDY225 and STUDY235). All participants provided written informed consent, and the study was prospectively registered in the German Clinical Trial Registry (DRKS00027643 for campaigns 1 and 2; DRKS00030848 for campaigns 3 and 4).

### 2.2 Participants and countermeasures

We accessed blood samples from 40 participants (18 women, age 35.1 ± 7.8 years, 22 men, age 36.2 ± 9.2 years). Retrospective analysis was performed on 16 samples from the LBNP and upright sitting groups, which were available after the completion of campaigns 1 and 2. These represent a subset of the originally enrolled participants (12 in the LBNP group and 11 in the upright sitting group) (Moestl et al., 2025). Participants were assigned to one of four groups during HDTBR: 8 participants in the LBNP group (n = 3 males, n = 5 females), 12 participants in the cycling and thigh cuff group (n = 8 males, n = 4 females), 8 participants in the upright sitting group (n = 4 males, n = 4 females), and 12 participants in the control group without countermeasures (n = 7 males, n = 5 females).

### 2.3 Clinical assessments

We performed general medical and full neurological examinations, including medical history (medication use, allergies, recent injuries) at the beginning, during HDTBR and at the end of the study. Daily ward rounds ensured continuous health monitoring.

### 2.4 Neuronal and glial biomarkers

We assessed biomarkers in serum (cycling with subsequent venous thigh cuff countermeasure group and control group) or plasma (LBNP and upright sitting control group) using the respective SIMOA Quanterix kits for NfL, GFAP, Aβ_40_, Aβ_42_, and total tau according to the manufacturer’s instructions (Li and Mielke, 2019). Samples were collected during a baseline data collection phase prior to HDTBR, at the end of HDTBR and during the recovery period after 13 days of HDTBR.

### 2.5 Statistics

Descriptive statistics were computed for demographic and anthropometric variables, such as age, and are reported as mean and standard deviation (SD). Mean deltas were calculated to illustrate changes in the biomarkers between the three time points: baseline, end HDTBR, and recovery. Differences were tested using paired Wilcoxon signed-rank tests. In addition, generalized linear mixed-effects models (GLMM) were estimated to examine the effects of time (wave: baseline, end HDTBR, recovery), intervention group (LBNP, cycling + cuffs, upright sitting, control), and their interaction on individual biomarker levels. Models included gender as a covariate and random intercepts for each participant to account for repeated measures. Estimated marginal means, pairwise contrasts, and model-based predictions with 95% confidence intervals were computed. Statistical significance was defined as p < 0.05. All analyses were conducted using R (version 4.4.2)^9^ using the packages ‘*lme4*’, ‘*emmeans*’, and ‘*ggpredict*’.

## 3 Results

**FIGURE 1:**
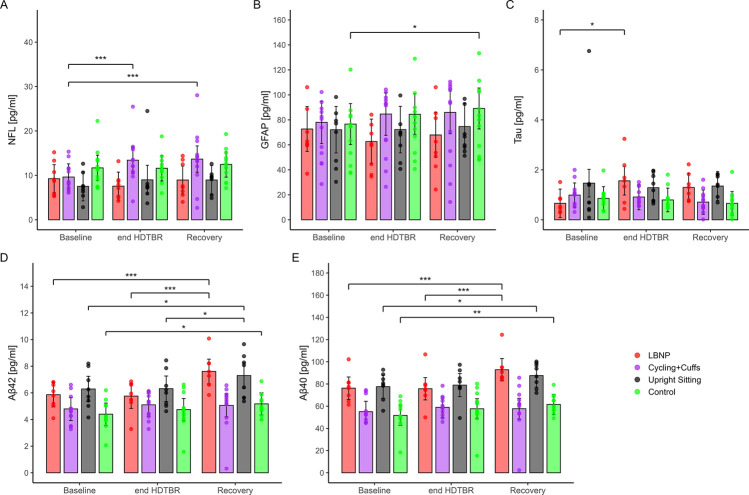
Biomarker assessment during HDTBR for **(A)** Neurofilament light chain (NfL) **(B)** Glial fibrillary acidic protein (GFAP) **(C)** Total Tau **(D)** β-Amyloid 1-42 (Aβ_42_) **(E)** β-Amyloid 1-40 (Aβ_40_). HDTBR = Head Down Tilt Bed Rest, LBNP = Lower Body Negative Pressure.

We observed no neurological deficits indicative of CNS impairment. Intermittent rotational vertigo was predominantly reported in the control group, with eight participants describing such symptoms (Moestl et al., 2025). While some participants reported vertigo, there were no indications of central vertigo. Although no clinical deficits were detected, subtle neuronal or glial changes might still be reflected in blood biomarkers. Therefore, we analyzed NfL, GFAP, Aβ_40_, Aβ_42_, and total tau levels in peripheral blood. Descriptive results revealed that the most pronounced biomarker increases from baseline to recovery for Aβ_40_ (+9.08 pg/mL) and Aβ_42_ (+0.86 pg/mL), followed by GFAP and NfL, while tau only showed small changes across time points (Table 1). The Aβ_42_/Aβ_40_ ratio remained stable across groups and time points with a ratio of 0.1. Multivariable GLMMs revealed several significant within-group changes over time. In the LBNP group, levels of Aβ_40_ (*p* < 0.001) and Aβ_42_ (*p* < 0.001) increased markedly from baseline to recovery (Figure 1). Estimated means rose from 76.9 to 93.5 pg/mL for Aβ_40_ and from 5.79 to 7.54 pg/mL for Aβ_42_, corresponding to deltas of +16.6 and +1.75 pg/mL, respectively. Aβ_42_ also showed smaller but significant increases in the upright sitting (Δ = +1.01 pg/mL, *p* = 0.02) and control groups (Δ = +0.77 pg/mL, *p* = 0.03), while Aβ_40_ rose in upright sitting (Δ = +9.95 pg/mL, *p* = 0.045) and control groups (Δ = +9.88 pg/mL, *p* = 0.01) from baseline to recovery (Figure 1). For NfL, a marker for axonal damage, the cycling and cuffs group showed strong increases from baseline to end HDTBR (Δ = +3.79 pg/mL, *p* < 0.001) and to recovery (Δ = +4.04 pg/mL, *p* < 0.001, Figure 1). Other groups exhibited no significant within-group changes. Regarding GFAP, only the control group showed a significant increase from baseline to recovery (Δ = +12.5 pg/mL, *p* = 0.01, Figure 1), while other contrasts were not significant. For tau, a modest increase was observed in the LBNP group from baseline to end HDTBR (Δ = +0.90 pg/mL, *p* = 0.03), though this effect did not persist into the recovery phase (Figure 1).

**TABLE 1:** Levels and differences in biomarkers at and between baseline, end of HDTBR, and recovery.

Biomarker	Baseline	end HDTBR	Recovery	Δ recovery - baseline	Δ end HDTBR - baseline	Δ recovery – end HDTBR
NfL	9.70 (3.76)	10.77 (4.92)	11.37 (4.87)	1.67 (3.70)	1.07 (4.04)	0.60 (3.14)
GFAP	68.70 (22.33)	71.07 (23.58)	74.38 (27.85)	5.67 (17.35)	2.36 (15.30)	3.31 (13.07)
Tau	1.01 (1.05)	1.10 (0.61)	0.95 (0.56)	−0.08 (1.10)	0.09 (1.05)	−0.14 (0.60)
Aβ_42_	5.11 (1.33)	5.29 (1.31)	5.97 (1.78)	0.86 (1.29)	0.18 (0.71)	0.68 (1.19)
Aβ_40_	63.47 (16.60)	66.60 (16.46)	72.55 (20.57)	9.08 (13.44)	3.12 (8.61)	5.96 (13.77)

Measurements are displayed as mean (standard deviation) at each of the three study phases. Individual changes (deltas) between timepoints display temporal trends. NFL = neurofilament light chain, GFAP = glial fibrillary acidic protein.

## 4 Discussion

The important finding of our study is that the LBNP group exhibited robust amyloid-related biomarker increases during HDTBR which were still present in the recovery period. In our study, descriptive statistics provided an initial overview of biomarker trajectories across timepoints and groups. These summaries did not indicate major changes in the biomarkers besides amyloid when comparing raw mean values from baseline to the different study phases. However, when applying a GLMM, which accounts for individual-level changes over time, within-subject correlations, and potential confounding variables like gender, more nuanced patterns emerged. Specifically, axonal markers like NfL levels increased significantly in the cycling and cuffs group throughout the study. In addition, tau protein levels also rose progressively in the LBNP group, but importantly, returned to baseline levels in the recovery phase, which may indicate reversibility and absence of sustained neuronal injury. In the control group, GFAP levels also showed a gradual and statistically significant increase throughout the study phases, suggesting low-level glial activation. However, we observed no clinical signs or symptoms of overt neurological impairment.

Established reference values and clinical interpretation algorithms for CSF-derived total tau, phospho-tau, and amyloid beta vary depending on the company’s reference standards (Lewczuk et al., 2009). To place our blood-based biomarker results in context and allow comparison with existing literature, we considered established reference values. The Aβ_42_/Aβ_40_ ratio remained stable across all groups and time points at 0.1, positioning it at the upper limit of the plasma reference range (0.053–0.098) reported in population-based studies of individuals aged 3–55 years (Cooper et al., 2023). Plasma levels of Aβ_42_ and Aβ_40_ in our study were below reported thresholds of 2.72–11.09 pg/mL and 61.4–303.9 pg/mL, respectively for older healthy individuals above age 50 (Chen et al., 2023). Mean NfL plasma concentrations were below the upper end of the normal reference range of 10 pg/mL for healthy individuals at the age of 18–50 years (Simrén et al., 2022) or 18 pg/mL in population-based samples up to 60 years old (Cooper et al., 2023). Mean NfL serum concentrations were below the upper end of the normal reference range of 21.4 pg/mL for healthy individuals at the age of 18–65 years (Hviid et al., 2020). Mean GFAP plasma and serum concentrations were also within the normal reference range (Cooper et al., 2023; Tybirk et al., 2022). Similarly, plasma tau concentrations were also well within the reported reference range for healthy individuals >50 years of 0.20–3.12 pg/mL^12^.

Evidence regarding the clinical utility of serum or plasma CNS biomarker analysis is still limited (Kac et al., 2022). Nevertheless, NfL is already used for monitoring disease progression and treatment response in, e. g., patients with Multiple Sclerosis (Hviid et al., 2022; Benkert et al., 2022). Extended space missions have been associated with increased levels of NfL and tau, possibly indicating neuronal damage, and elevated GFAP levels, implying astrocyte activation (Eulenburg et al., 2021). In contrast, repeated bouts of micro- and hypergravity during parabolic flights showed elevations of GFAP and S100B suggesting glio-vascular damage, but biomarkers of neuronal-axonal damage (NfL, ubiquitin carboxy-terminal hydrolase L1 (UCH-L1) and total tau) in serum did not change (Bailey et al., 2020). On contrast, 14 days of continuous HDTBR in older individuals aged >50 years demonstrated increased serum levels of NfL, GFAP, tumor necrosis factor alpha (TNF-α), and interleukin-6 (IL-6) with no intervention (exercise countermeasure) (Blaber et al., 2023). No effects were observed for total tau, myelin basic protein, or UCH-L1 (Blaber et al., 2023). Exposure to high G-forces during spaceflight launch and landing could also impact brain physiology. In aviators, high G-forces amplify brain hypoperfusion and oxidative stress, disrupting cellular metabolism and promoting pathological protein production (Rhind et al., 2024). Canadian Armed Forces aviators exhibited significantly elevated plasma levels of GFAP, NfL, PRDX-6 (marker for oxidative stress), and total tau compared to controls, indicating axonal and glial damage (Rhind et al., 2024).

In general, spaceflight induces an upward brain shift, grey matter redistribution, CSF flow alterations, and ventricular expansion (Seidler et al., 2024) likely hindering metabolite removal through the glymphatic system (Miller et al., 2022; Wostyn et al., 2020). Together with other conditions like radiation exposure, confinement and sleep disturbances, this could disrupt fluid dynamics, alter metabolic processes, and impair the efficient removal of neurotoxic waste, potentially increasing the risk of neurodegenerative changes (Miller et al., 2022). For example, an increased risk of Parkinson’s disease (PD) incidence was observed in association with prolonged occupational exposure to external gamma radiation, particularly at cumulative doses exceeding 0.1 Gy (Azizova et al., 2020). The risk estimate rose progressively with higher radiation doses (Azizova et al., 2020).

Emerging research suggests that glymphatic system impairment plays a central role in neurodegenerative diseases such as Alzheimer’s and Parkinson’s, by reduced clearance of toxic proteins like Aβ, tau and α-synuclein from the brain (Sun et al., 2025). Research indicates that astronauts may experience parkinsonian symptoms due to systemic mitochondrial dysfunction and dopamine loss (Ali et al., 2025). It has been estimated that direct transport of Aβ across the blood-brain barrier accounts for ∼25% of Aβ clearance (Roberts et al., 2014). Nevertheless, the Aβ levels presented in this study were still lower in all groups compared to elderly (>60 years old) healthy controls or patients with Alzheimer’s disease (Janelidze et al., 2016). In these cohorts mean Aβ_42_ levels ranged from 13 to 20 pg/mL and Aβ_40_ from 239 to 284 pg/ml (Janelidze et al., 2016). In this context, LBNP may aid in metabolite clearance, as we observed an improved washout of Aβ after simulated microgravity similar to studies in cosmonauts after spaceflight (Eulenburg et al., 2021). We know that structural changes after spaceflight in the brain are subject to a prolonged and incomplete recovery processes (van Ombergen et al., 2019). Mitigating or preventing pathological CNS changes, such as CSF flow alterations and brain shift, through countermeasures like LBNP in (simulated) microgravity could therefore prevent or ameliorate the CNS changes and further enhance clearance of proteins during recovery periods. The effectiveness of LBNP in our study, however, was limited by the application of −25 mmHg due to concerns about pre-syncopal symptoms. In contrast, a novel mobile gravity suit, designed to be compact, untethered, and flexible for improved mobility in space, was well tolerated at pressures as low as −40 mmHg (Ashari and Hargens, 2020). This suit generates greater ground reaction forces compared to a standard LBNP chamber, likely due to its innovative design, which enables a higher percentage of body weight support (Ashari and Hargens, 2020).

In line with these results, animal studies showed that simulated microgravity and space radiation can significantly impair brain health. In space-flown mice, Masarapu et al. applied spatial multiomics to reveal region-specific disruptions in neurogenesis, synaptic function, and immune regulation, alongside oxidative stress (Masarapu et al., 2024). Pathway analysis revealed protein misfolding and impaired clearance mechanisms, suggesting similarities to neurodegenerative diseases characterized by pathological protein accumulation (Masarapu et al., 2024). Mao et al. confirmed that actual spaceflight to the International Space Station damages blood-brain barrier integrity, with increased apoptosis in the hippocampus and altered expression of tight junction and endothelial markers in mice (Mao et al., 2020). Ground-based models further support these findings: combined hindlimb unloading (HU) and low-dose radiation (LDR) or photon irradiation caused oxidative brain damage, reduced antioxidant defense and resulted in metabolic alterations (Mao et al., 2016; Raber et al., 2023). Overbey et al. found that HU and LDR in mice leads to long-term transcriptional and epigenetic changes in pathways regulating neuroplasticity and neurogenesis (Overbey et al., 2019). In addition, in swine, Iacono et al. detected radiation-induced proteomic shifts in the hippocampus, including upregulation of neuroprotective proteins (Iacono et al., 2024).

The identification of neuronal and glial biomarkers is therefore crucial for early detection and intervention in these neurodegenerative diseases, with ongoing studies exploring neuroprotective strategies and therapeutic targets that could mitigate space-induced neuropathology. In addition, future studies are needed to define a common cut-off for the definition of pathologic states independent of the material used.

This study has several limitations. The number of participants per campaign was limited to 12 due to the availability of single-occupancy rooms at the study facility, which restricted overall sample size. The primary objective was to investigate SANS-related changes; the study was not designed or powered to detect sex-specific effects, even though both sexes were included. Additionally, some retrospective analyses were limited to a subset of 16 available samples from the LBNP and upright sitting groups, which further constrains subgroup (gender-specific) analyses and generalizability.

Although CSF is considered the gold standard for assessing CNS pathology, its use in spaceflight and analog studies, as for this study, is limited by the invasiveness of lumbar puncture and the need for trained personnel. Consequently, blood-based biomarkers offer a practical alternative. While head-to-head comparisons between CSF and blood are lacking in bed rest studies, several lines of evidence support the utility of blood-based markers. For example, NfL levels in plasma and CSF are generally well correlated and show comparable discriminatory power for neurodegenerative conditions such as PD and atypical PD, despite slightly higher diagnostic accuracy in CSF (Baiardi et al., 2025). In contrast, tau measures show poor correlation between plasma and CSF, highlighting that not all markers translate equally across compartments (Fossati et al., 2019). Interestingly, plasma GFAP has shown stronger associations with amyloid pathology and larger group differences than CSF GFAP (Benedet et al., 2021). In addition, both ELISA and Simoa assays have shown reliable performance in detecting cerebral amyloidosis through plasma Aβ_42_/Aβ_40_ ratios, with high negative predictive values (Meyer et al., 2024). Advances in ultrasensitive assays now allow reliable quantification of key markers such as p-tau181, p-tau217, NfL, and GFAP in blood, with good predictive performance for neurodegenerative diseases causing dementia (Grande et al., 2025). Nonetheless, systematic head-to-head comparisons between CSF and blood biomarkers are critically needed to strengthen confidence in blood-based assessments of CNS pathology. Overall, the choice between serum and plasma as a source material for CNS biomarker analysis is a critical factor in study design and data interpretation, particularly for group comparisons between countermeasures and control groups. While both originate from blood, they differ in preparation and composition, which can influence biomarker quantification and comparability across studies (Huebschmann et al., 2020). In contrast to other studies, we detected lower NfL levels in serum than in plasma (Kwon et al., 2023) and not higher GFAP levels in serum (Huebschmann et al., 2020). While confounding factors such as vascular comorbidities and medication use for patients must be considered, the analytical and clinical validity of blood-based biomarkers is increasingly supported, making them a valuable tool for monitoring CNS-related changes in spaceflight and analog research (Schöll et al., 2024).

Overall, 30-day HDTBR as a spaceflight analog can reproduce changes in circulating CNS biomarkers previously observed in long-duration space missions. These findings are particularly relevant: even minor, subclinical neurobiological changes may accumulate during extended missions, and detecting them early could inform countermeasure strategies and monitoring protocols.

## Data Availability

The raw data supporting the conclusions of this article will be made available by the authors, without undue reservation.
